# Relapsing viral keratoconjunctivitis in COVID-19: a case report

**DOI:** 10.1186/s12985-020-01370-6

**Published:** 2020-07-08

**Authors:** Dongyu Guo, Jianhua Xia, Yang Wang, Xuhong Zhang, Ye Shen, Jian-Ping Tong

**Affiliations:** Department of Ophthalmology, Zhejiang University School of Medicine First Affiliated Hospital, Hangzhou, 310003 Zhejiang China

**Keywords:** Coronavirus, SARS-CoV-2, COVID-19, Conjunctivitis, Cytokine surge

## Abstract

**Background:**

Since the outbreak of Coronavirus Disease 2019 (COVID-19) in December 2019, many studies have reported the presence of severe acute respiratory syndrome coronavirus 2 (SARS-CoV-2) in the conjunctival sac of patients infected with this virus, with several patients displaying symptoms of viral conjunctivitis. However, to our best knowledge, there is no in-depth report on the course of patients with COVID-19 complicated by relapsing viral conjunctivitis or keratoconjunctivitis.

**Case presentation:**

A 53-year-old man confirmed with COVID-19 developed symptoms of viral conjunctivitis in the left eye approximately 10 days after the onset of COVID-19. The results of a nucleic acid test were positive for SARS-CoV-2 in the conjunctival sac of the left eye. The symptoms were relieved 6 days after treatment. However, the patient was subsequently diagnosed with viral keratoconjunctivitis in both eyes 5 days after the symptoms in the left eye were satisfactorily relieved. The disease progressed rapidly, with spot staining observed at the periphery of the corneal epithelium. Although SARS-CoV-2 could not be detected in conjunctival secretions, the levels of inflammatory factors, such as interleukin-6, were increased in both eyes. Both eyes were treated with glucocorticoids, and symptoms were controlled within 5 days. There was no recurrence.

**Conclusions:**

In this case report, the pathogenesis, clinical manifestations, treatment, and outcome of a case with COVID-19 complicated by relapsing viral keratoconjunctivitis is described, and the involvement of topical cytokine surge in the pathogenesis of COVID-19 as it relates to viral keratoconjunctivitis is reported.

## Background

Since the outbreak of Coronavirus Disease 2019 (COVID-19) in December 2019, the number of infections and deaths has increased exponentially. At the time of this writing, more than 2 million people have been infected with SARS-CoV-2 worldwide [1]. On 11 February 2020, the disease caused by the novel coronavirus was designated COVID-19 by the World Health Organization [2], and the causative pathogen was identified as severe acute respiratory syndrome coronavirus 2 (SARS-CoV-2) by the International Committee on Taxonomy of Viruses [3]. SARS-CoV-2 is the seventh species of β-coronavirus to infect humans, with 85% homology to bat-SL-CoVZC45 [4].

In February 2020, Lu et al. suggested that the ocular surface might be a route of SARS-CoV-2 transmission, and studies on the association between the virus and the ocular surface were initiated [5]. In March 2020, Deng et al. reported that SARS-CoV-2 can infect rhesus macaques through the conjunctiva [6]. Subsequently, Zhou et al. demonstrated the presence of SARS-CoV-2 in the conjunctival sac of patients with COVID-19 [7–9]. In addition, Chen et al. reported an association between conjunctival congestion, the main COVID-19-related ocular symptom, and conjunctivitis, suggesting that SARS-CoV-2 can cause conjunctivitis [10].

At the time of this writing, there is only one case report on the course of a patient with COVID-19 complicated by viral conjunctivitis [11], and the pathogenesis of SARS-CoV-2-related conjunctivitis was not sufficiently addressed in their case report. In view of the ongoing COVID-19 pandemic, a recent study has reported elevated inflammatory factor levels in severely ill patients, indicating that cytokine surge is involved in the pathogenesis of COVID-19 [12]. However, the significance of topical cytokine surge in SARS-CoV-2-related conjunctivitis/keratitis is unknown. Therefore, the pathogenesis, clinical manifestations, treatment, and outcome of a case with COVID-19 complicated by relapsing viral keratoconjunctivitis are shared here, thus providing a guidance for future research.

## Case presentation

A male patient, 53 years old, was admitted to the First Affiliated Hospital of Zhejiang University on 8 February 2020 for a history of 12-day dry cough, 6-day fever with a maximum body temperature of 38.0°C, and 3-day disease aggravation with shortness of breath. Three days before admission, nasopharyngeal swab specimens were obtained and tested for SARS-CoV-2; the results of nucleic acid tests were positive twice. Thoracic computed tomography (CT) scans showed thin exudate in both lungs. The patient was diagnosed with COVID-19 and transferred to our hospital.

**Fig. 1:**
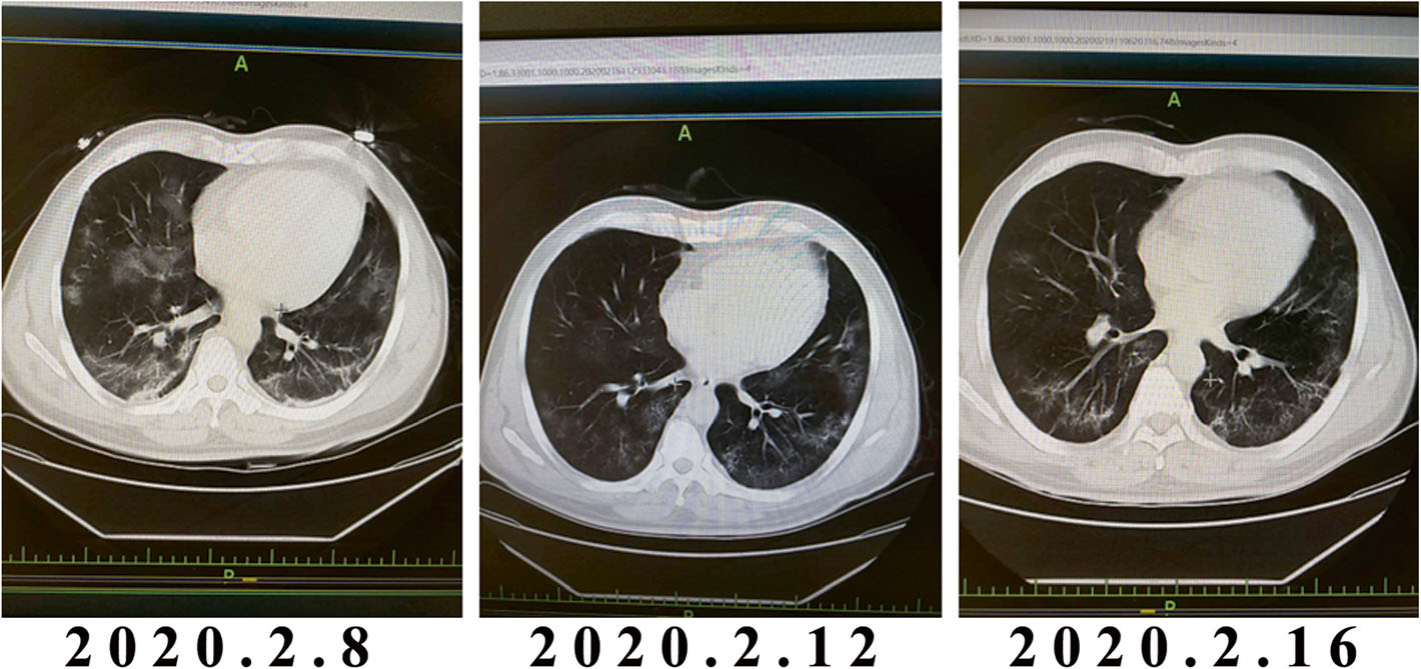
Thoracic CT images of the patient

The body temperature was 36.6°C and it remained normal after admission. A CT scan indicated extensive inflammation in both lungs (Fig. 1, left panel), and no conjunctivitis was noted in neither eye. The patient was treated with oral arbidol (200 mg, tid), recombinant interferon spray (100,000 U, qid), oral darunavir (800mg, qd) as well as oxygen and other symptomatic supportive treatments. A CT scan revealed improvement (Fig. 1, middle and right panels), and there were no other complaints.

**Fig. 2:**
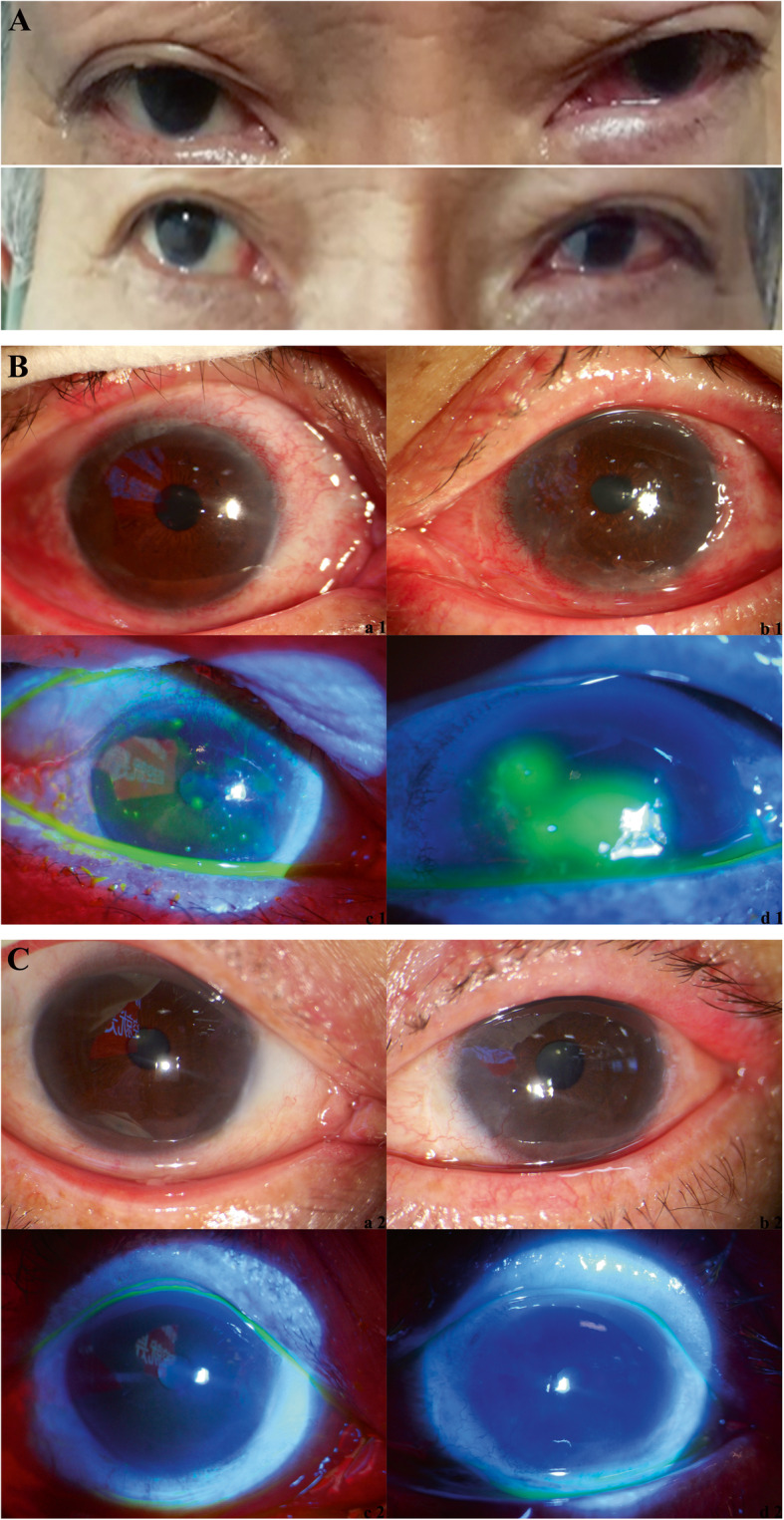
Eye images of the patients. **a** Image taken on 16 February 2020. **b** Slit lamp images taken on 1 March 2020. Panels (a1) and (c1) show the right eye and (c1) shows the right eye stained with fluorescein sodium and visualized under a cobalt blue light. Panels (b1) and (d1) show the left eye and (d1) shows the left eye stained with fluorescein sodium and visualized under a cobalt blue light. **c** Slit lamp images taken on 8 March 2020. Panels (a2) and (c2) show the right eye, and (c2) shows the right eye stained with fluorescein sodium and visualized under a cobalt blue light. Panels (b2) and (d2) show the left eye, and (d2) shows the left eye stained with fluorescein sodium and visualized under a cobalt blue light.

On 16 February 2020, the patient complained of general discomfort and stabbing pain in the left eye. And he did not report visual loss in either eye. An eye examination revealed redness and edema of lower eyelid, bulbar conjunctival hyperemia and edema, accompanied by a large quantity of watery secretions and a small quantity of thin viscous secretions. The cornea was clear, without infiltration. The right eye was relatively normal, except that slight redness and edema of the lower eyelid were obvious (Fig. 2a). On 17 February 2020, binocular tear and conjunctival secretion specimens were obtained using swabs and tested for SARS-CoV-2. The results of nucleic acid tests were positive (for details, see Reference 8 [8]); specifically, the left eye was positive for the virus, whereas the right eye was negative. The patient stated that he had a tendency of not washing his hands and rubbing his eyes, and he was advised to reverse his bad habit. On 18 February 2020, the left eye was treated with levofloxacin hydrochloride (drops, tid) and 0.1% sodium hyaluronate (drops, tid). One day after treatment, specimens were obtained and tested as indicated, and the left eye was positive for the virus, whereas the right eye was negative. On 22 February 2020, the symptoms in the left eye subsided. The results of nucleic acid tests using left eye tear and conjunctival secretion specimens, as well as sputum and fecal specimes, were all negative for SARS-CoV-2. From 19 to 23 February 2020, left eye tears and conjunctival secretion specimens were repeatedly obtained for virus isolation and culture, and the results were all negative. The treatment of the left eye was halted, and the patient was discharged and quarantined at home.

**Figure 3:**
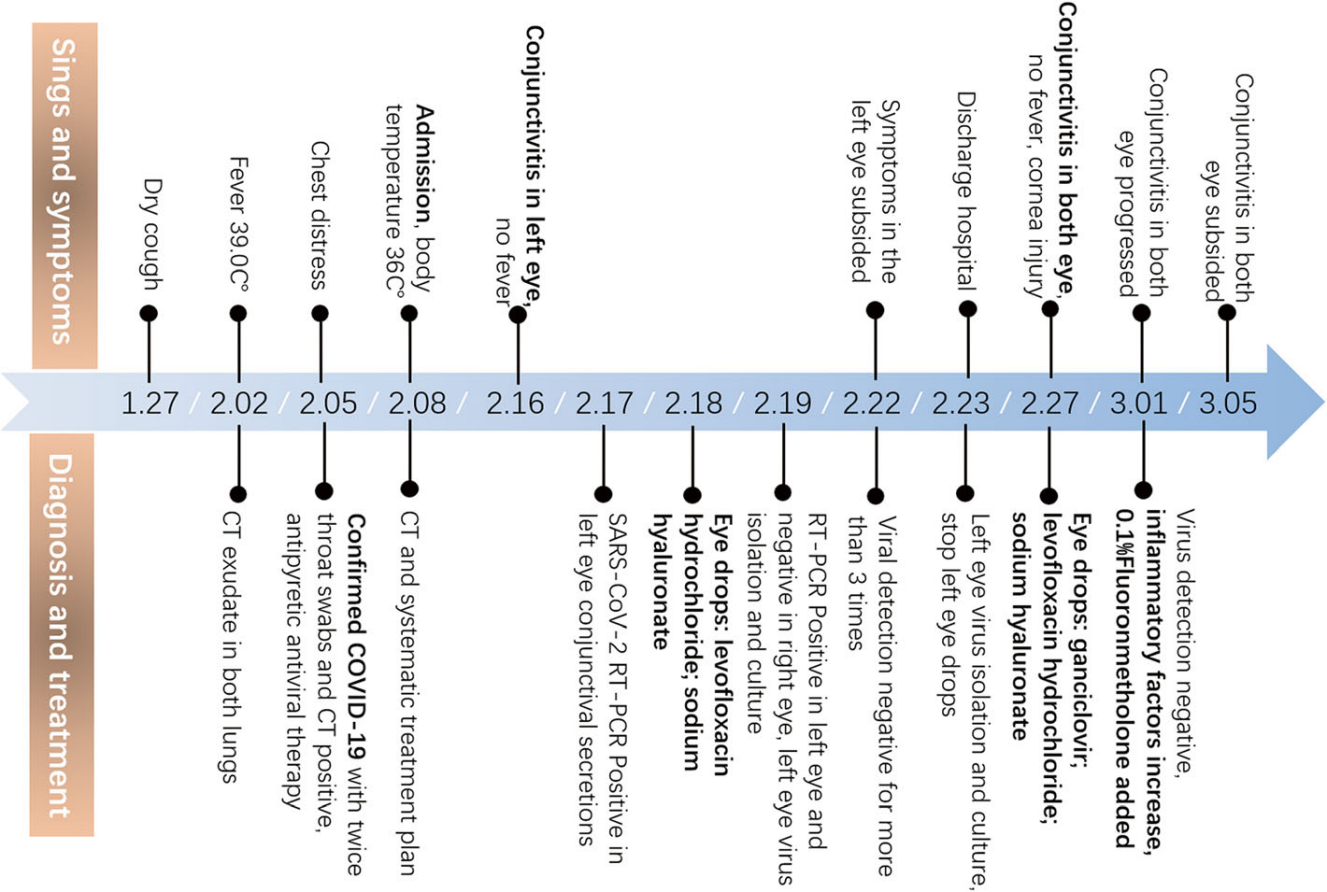
Timeline of the disease course

On 27 February 2020, the patient phoned the clinic and stated that he had sudden discomfort in both eyes with conjunctival hyperemia and edema. However, the patient denied recently rubbing his eyes. Both eyes were treated with ganciclovir ophthalmic gel, levofloxacin hydrochloride drops, and 0.1% sodium hyaluronate drops. On 1 March 2020, the patient was re-examined. However, the symptoms of both eyes were not relieved; instead, they were aggravated, with spot staining observed at the periphery of the corneal epithelium of both eyes (Fig. 2b). Binocular tear and conjunctival secretion specimens were obtained and tested for SARS-CoV-2, adenovirus, and herpes simplex, as well as for inflammatory factors. The results of nucleic acid tests were all negative for the viruses; however, the levels of inflammatory factors, such as interleukin-6 (IL-6), increased ten-fold of the upper normal reference range in the left eye (Table 1). Both eyes were treated with 0.1% fluorometholone (drops, qid). Four days after treatment, binocular symptoms were relieved. On 8 March 2020, the patient was re-examined. Binocular keratoconjunctivitis was completely relieved, and the cornea was transparent (Fig. 2c). The timeline of the disease course is shown in Fig. 3.

**Table 1:** The inflammatory factors in conjunctival secretions and blood

	Left eye	Right eye	Normal reference range in conjunctival secretions	Blood	Normal reference range in blood
IL-2 (pg/ml)	<1.64	<1.64	<0.71	<1.10	0.00–4.13
IL-4 (pg/ml)	<1.05	<1.05	<1.03	<1.84	0.00–8.37
IL-6 (pg/ml)	74.91	9.24	<1.34	22.14	0.00–6.61
IL-10 (pg/ml)	<1.09	<1.09	<0.66	3.94	0.00–2.31
TNF-a (pg/ml)	<1.34	<1.34	<6.71	17.10	0.00–33.27
TNF-g (pg/ml)	0.74	0.92	<4.07	5.36	0.00–20.06

## Discussion and conclusions

In this case report, the diagnosis, disease course, and treatment of a patient with COVID-19 complicated by relapsing viral keratoconjunctivitis are described. The patient developed symptoms of conjunctivitis in the left eye approximately 10 days after the onset of COVID-19. The results of nucleic acid tests were positive for SARS-CoV-2 in the conjunctival sac of the left eye. However, the results of tests for other common viruses that cause conjunctivitis, such as adenovirus and herpes simplex virus, were all negative. Therefore, we concluded that viral conjunctivitis was related to SARS-CoV-2 infection in this case.

In this patient, the main symptom of conjunctivitis were general discomfort and stabbing pain. The typical symptoms of conjunctivitis are bulbar conjunctiva congestion and edema, accompanied by a large quantity of watery secretions and a small quantity of thin viscous secretions, an absence of follicles or pseudo-membranes formation, a lack of the corneal infiltration at the initial stage of infection, no decrease in vision, and no swelling of the lymph nodes near the anterior and posterior ear. At late stages of infection, the symptoms of conjunctivitis were similar with those at early stages, and corneal infection involved peripheral infiltration with epithelial involvement.

In this patient, the initial course of conjunctivitis was less than 1 week. The patient consistently wore a mask, although the upper edges of the mask did not fit closely with the nose root. Therefore, it is very likely that infectious respiratory droplets entered the eyes. In addition, the patient had a bad habit of eye rubbing, which led us to speculate that the conjunctival infection was due to external factors. In addition, the results of repeated nucleic acid tests revealed that the virus could not exist in the conjunctival sac for a long time, because it could not be detected in the conjunctival sac after three days, and these results are consistent with those of Chen et al. [10]. However, we could not rule out the presence of residual virus in the conjunctival sac at late stages of the infection, because conjunctival secretion specimens are hard to obtain, quantities are little also, which results in the relatively high specificity and low sensitivity of the polymerase chain reaction assays method [13]. The initial progression of conjunctivitis might have been related to the proliferation rate of SARS-CoV-2 in the conjunctival sac. SARS-CoV-2 infection requires binding to the angiotensin converting enzyme 2 (ACE2) receptor [14, 15]. ACE2 is widely expressed by a variety of cells in the human body, and its expression is highest in pulmonary capillary endothelial cells. Another study has also reported ACE2 expression in the human conjunctiva [16], suggesting that SARS-CoV-2 might exist in conjunctival secretions. In our patient, conjunctival secretion specimens were repeatedly obtained for virus isolation and culture, but the results were negative, indicating that SARS-CoV-2 might not proliferate in the conjunctiva and cause conjunctivitis through direct invasion and destruction.

Symptoms of viral keratoconjunctivitis re-appeared in both eyes approximately 5 days after the symptoms in the left eye were satisfactorily relieved. Symptoms were similar to those at early stages of infection, except that disease progressed more rapidly and corneal involvement at the periphery of the cornea was obvious, which was indicative of reactive disease. The results of nucleic acid tests for related viruses in the conjunctival sac were all negative, although these findings might have been caused by a history of topical antiviral drug use. However, the levels of a variety inflammatory factors increased significantly. Therefore, we speculate that the disease was not caused by the direct invasion and destruction of SARS-CoV-2, but rather "topical cytokine surge" caused by the autoimmune response.

Cytokine and chemokine responses generated against viruses play important roles in mediating crosstalk among the various immune cells and in connecting innate and adaptive immunity. Upon infection, cytokine levels are elevated. Subsequently, inducible inflammatory chemokines direct effector leukocytes to specific tissues, which can upset the immune regulatory network through the constant self-amplification of the positive feedback loop, thereby resulting in cytokine surge [17, 18]. A recent study has reported that the levels of inflammatory factors, such as G-CSF, TNF-α, IL-6, and IP-10, are significantly higher in severely ill COVID-19 patients than those in less severely ill patients, indicating that cytokine surge is involved in the pathogenesis of the disease [12]. In infectious immune corneal injuries, bilateral peripheral ulcerative keratitis caused by the herpes simplex virus [19] and bilateral disciform keratitis complicated by adenoviral conjunctivitis [20] due to the induction of inflammatory reactions or immunological responses in the corneal stroma by immune-degrading complexes have also been reported. However, these delayed corneal infiltrations display different clinical signs, and therefore, they are often misdiagnosed. In this patient, the clinical signs of peripheral infiltration with epithelial involvement were similar to those of viral infection; however, this may have been caused by the presence of different immune cells within tissues [21]. Based on the findings of the aforementioned studies, we conclude that viral infections of the eyes, together with increased levels of serum cytokines, tend to associate with corneal infiltration with epithelial involvement or ulceration, resulting in the recruitment of NK cells. On the other hand, viral infections of the eyes tend to associate with corneal infiltration with stromal involvement, CD4+ T cell reactivity [22], and antigen deposition [23]. In this patient, the serum IL-6 level was two-fold higher than the normal reference range 4 days after hospital discharge. This pro-inflammatory cytokine can diffuse into the conjunctiva, which is rich in blood vessels. In addition, SARS-CoV-2-infected epithelial cells of the conjunctiva may express inflammatory chemokines, thereby recruiting more inflammatory cells and producing more pro-inflammatory cytokines [24]. As a result, the IL-6 level in the left eye was much higher than that in the right eye, and the IL-6 level in the right eye was similar to that in the blood. Therefore, we conclude that conjunctivitis or keratoconjunctivitis caused by SARS-CoV-2, especially the symptoms at late stages (7 days or later) of the infection, was not due to the local invasion and destruction of the virus; instead, it was due to cytokine surge. The extensive pro-inflammatory cytokine and chemokine response caused by the virus in epithelial cells of the conjunctiva and corneal limbus eventually resulted in apoptosis of epithelial cells of the peripheral cornea and reactive inflammatory injury rather than apoptosis of epithelial cells of the central cornea. This might explain why the early stages of the infection involved only one eye, whereas cytokine surge involved both eyes.

According to the treatment guidelines of epithelial keratitis, glucocorticoids are not usually recommended for punctiform viral epithelial keratitis, because they are believed to promote viral replication. However, in this case, cytokine surge, rather than viral invasion, played a major role. Therefore, glucocorticoid could successfully control symptoms within 5 days, with no recurrence, indicating that hormone therapy was effective, although the symptoms involves corneal epithelial defects. In addition, our findings indicated that cytokine surge might play an important role in the delayed aggravation. In cases of severe corneal inflammation, it might be feasible to inhibit IL-6 production [25], because this cytokine has important roles in inflammation.

Our findings indicated that SARS-CoV-2 could induce viral conjunctivitis through an exogenous mechanism. At early stages of the disease, local invasion and inflammation contributed to the localized nature of the disease, and the disease course did not exceed 1 week. At late stages of the disease, keratoconjunctivitis was aggravated and more widespread, which was related to cytokine surge. Therefore, we conclude that aggressive medicinal intervention, longer and more frequent follow-up, and proper use of topical glucocorticoids can diminish cytokine surge.

## Data Availability

The datasets used and/or analyzed in this study are available from the corresponding authors upon reasonable request.

## Abbreviations

COVID-19: Coronavirus disease 2019
SARS-CoV-2: Severe acute respiratory syndrome coronavirus 2
IL: Interleukin
bat-SL-CoVZC45: Bat-derived SARS-like coronavirus isolate bat-SL-CoVZC45
CT: Computed tomography
qd: One time a day
tid: Three times a day
qid: Four times a day
ACE: Angiotensin converting enzyme
G-CSF: Granulocyte colony stimulating factor
TNF: Tumor necrosis factor
IP-10: Interferon-induced protein 10
